# Thermal Diffusivity and Conductivity of Polyolefins by Thermal Lens Technique

**DOI:** 10.3390/polym14132707

**Published:** 2022-07-01

**Authors:** Behnaz Abbasgholi-NA, Seyed Reza Nokhbeh, Osamah A. Aldaghri, Khalid Hassan Ibnaouf, Nawal Madkhali, Humberto Cabrera

**Affiliations:** 1 Optics Lab, STI Unit, The Abdus Salam International Centre for Theoretical Physics, 34151 Trieste, Italy; be.asbaghi@yahoo.com; 2 NanoInnovation Laboratory, Elettra-Sincrotrone Trieste S.C.p.A., 34151 Trieste, Italy; 3 Department of Chemistry, Ferdowsi University of Mashhad, Mashhad 9177948974, Iran; seyedrezanokhbeh@mail.um.ac.ir; 4 Physics Department, College of Sciences, Imam Mohammad Ibn Saud Islamic University (IMSIU), Riyadh 13318, Saudi Arabia; odaghri@imamu.edu.sa (O.A.A.); khiahmed@imamu.edu.sa (K.H.I.)

**Keywords:** thermal lens spectrometry, thermal diffusivity, thermal conductivity, long-chain branching number, polymers, polyolefin

## Abstract

A mode-mismatched thermal lens spectrometry (TLS) technique, in a pump–probe two-laser-beam configuration, was employed for the experimental determination of the thermal properties of four selected well-characterized polyolefin homopolymer films. We investigated the thermal diffusivity (D) and thermal conductivity (κ) of high-density polyethylene, low-density polyethylene, linear low-density polyethylene, and polypropylene. We also measured the structural properties (i.e., average molecular weight, polydispersity index, branching number), along with the rheological and thermal properties (i.e., melting point, specific heat capacity Cp, degree of crystallinity) of samples by high-temperature gel permeation chromatography (HT-GPC), rheometric mechanical spectrometry (RMS), differential scanning calorimetry (DSC), and densitometry. The relationship between microstructural properties such as degree of crystallinity, D, and κ was investigated. The results show that there is good correlation between the degree of crystallinity and D. The TL technique enables measurement of D in semitransparent thin films within an uncertainty of 4%.

## 1. Introduction

In recent years, attention has been paid to the development of new methods for determining the thermal properties of materials, including polymers [1,2,3]. Heat-transport properties—such as thermal diffusivity (D) and thermal conductivity (κ)—of polyolefins, as one of the most widely used groups of polymers, are crucial parameters to be considered according to the type of application [4,5].

To date, several techniques have been used for the determination of D and κ. These techniques include photoacoustic spectroscopy [6], transient short-hot-wire [7], laser flash [8], quadratic frequency-modulated thermal wave imaging [9], forced Rayleigh light scattering [10], modified Angstrom [11], infrared thermography [12], laser intensity modulation method (LIMM) [13], temperature wave using joule-heating [14], pulsed photothermal radiometry [15], mirage [16], pulsed electrothermal [17], and thermal lens spectrometry (TLS) [18].

The methods based on the photothermal phenomenon are considered powerful tools for non-destructive measurements of the thermal and optical properties of various materials. These techniques are highly sensitive because only the absorbed radiation contributes to the signal. TLS is a photothermal technique commonly used for the structural and thermo-optical characterization of semitransparent thin films [18,19]. The TL effect occurs when the energy absorbed from a Gaussian beam produces local heating within the absorbing medium around the beam axis. In such experiments, the sample is exposed to a laser beam that has a Gaussian profile, and it excites the molecules along the beam’s path. The thermal relaxation of the excited molecules dissipates heat into the surroundings, thereby creating a temperature distribution that produces a refractive index gradient within the medium. The refractive index gradient acts as a diverging lens, which is referred to as a thermal lens (TL).

Typically, in semitransparent and thin samples, the absorbance is negligible and we cannot use any of the abovementioned techniques because of their low sensitivity. On the other hand, the sensitivity can be increased using high excitation power, which also increases the photodamage and causes melting in polyolefin materials. For these reasons, a highly sensitive photothermal method could be a good option, because stronger TL signals are obtained even using low excitation power [19].

On the other hand, the behavior of the thermo-optical properties of any kind of material can be correlated with their mechanical and structural properties. Especially in polymers, the relatively small change in structure can be correlated with a reasonable change in the value of specific heat capacity (Cp). Other parameters, such as κ and D, are associated with the heat-transfer ability of the material, which depends on its structural properties. As a result, in the case of polymers, the thermal properties depend on many factors, such as chemical constituents, average molecular weight, crystallinity, molecular orientation type, processing conditions, and temperature [20,21,22].

In this work, the TLS technique was applied for the measurement of D in four widely used polyolefins, including high-density polyethylene (HDPE), low-density polyethylene (LDPE), linear low-density polyethylene (LLDPE), and polypropylene (PP). Moreover, the measured physical and structural properties (i.e., density ρ, molecular weight averages (M_P_, M_n_, M_w_, M_z_, and M_z+1_), molecular weight distribution, bulk intrinsic viscosity, polydispersity index, rheological parameters, Cp, melting point, crystallinity, and long-chain branching number) were investigated and correlated with the thermal properties.

## 2. Materials and Methods

### 2.1. Samples

Four different commercial grades of polyolefins were supplied as pellets from shamsjavid Co. Ltd. Tehran, Iran (Table 1). Polymer films were made from pellets using a hot press (Dr. Collin GMBH P400P, Maitenbeth, Germany) at a predetermined 180 and 195 °C for PEs and PP, respectively, at a pressure of 100 bar (10 MPa) for 3 min. The films were cut into pieces (2.5 × 2.5 cm). 1,2,4-Trichlorobenzene (TCB, ≥ 99%) and butylated hydroxytoluene (BHT, ≥ 99%) were purchased from Merck Co.

### 2.2. TLS Setup

**Figure 1:**
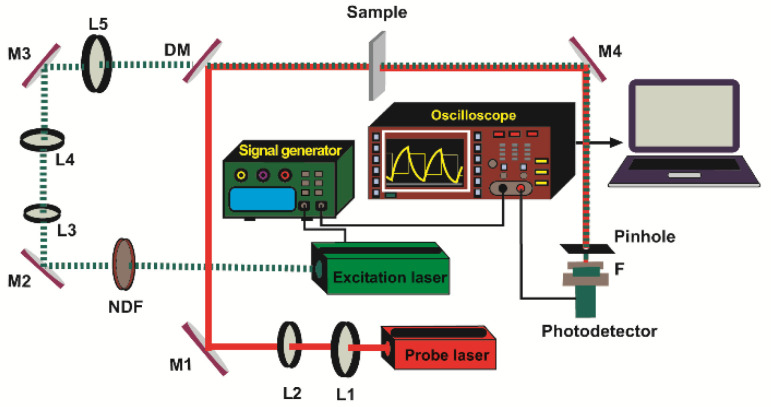
Scheme of the dual-beam mode-mismatched configuration. L1, L2, L3, L4, L5: lenses; M1, M2, M3, M4: turning mirrors; DM: dichroic mirror; NDF: neutral-density filter; F: filter.

In order to measure the D of the samples, we used the experimental setup shown in Figure 1. The probe beam was a 2 mW He-Ne laser (05-UR-111, Melles Griot, Carlsbad, CA, USA) collimated by the lenses L3 (LB1027-A, f = 40 mm, Thorlabs, Newton, NJ, USA) and L4 (LB1676-A, f = 100 mm, Thorlabs), respectively. A 532 nm diode-pumped solid-state laser (MGL-III- 532 nm-100, UltraLasers, Hewitt Cir, Newmarket, ON, Canada) excited the sample to produce the TL. Its output power was fixed at 80 mW by the neutral-density filter (NDF) (NDC 50S-3, Thorlabs, Newton, NJ, USA). The beam was modulated at 2 Hz using a signal generator (SG) (Rigol DG 2041A, Batronix, Preetz, Germany). The excitation beam was first collimated by the lenses L1 (LB1027-A, f = 40 mm, Thorlabs, Newton, NJ, USA) and L2 (LB1676-A, f = 100 mm, Thorlabs, Newton, NJ, USA), and then focused onto the sample by lens L3 (LB1676-A, f = 100 mm, Thorlabs, Newton, NJ, USA). The probe beam’s intensity changes were detected using a silicon detector (PDA 36A-EC, Thorlabs, Newton, NJ, USA) equipped with a 0.5 mm diameter pinhole. The TL signal was recorded and stored using a digital oscilloscope (RIGOL DS1102E, RIGOL Technologies, Inc., Beaverton, OR, USA). The 632.8 nm interference filter (MELLES GRIOT) removed any residual light at 532 nm.

The TLS signal processing and fitting procedure was performed using the theoretical model described in [23]. In this approach, the thermal lens signal S(*z*,*t*) is defined as the relative change in the light transmission T(*z*,t) through a small aperture in the presence of the excitation beam, and T(0) is its initial value when the excitation beam is off:

(1) $$S\left(z,t\right)=\frac{T\left(z,t\right)-T(0)}{T(0)}$$

In the situation of a small phase shift (<0.2) and small aperture radius (<1 mm), the signal can be written as follows [23]:

(2) $$S\left(z,t\right){=\Theta \tan}^{-1}\left\{\frac{4m(z)\nu (z)t/t_{c}\left(z\right)}{\left[1+2m\left(z\right)+\nu {\left(z\right)}^{2}\right]2t/t_{c}\left(z\right)+{\left[1+2m\left(z\right)\right]}^{2}{+\nu (z)}^{2}}\right\}$$

where t_c_ is the characteristic time of the thermal lens $t_{c}=\omega_{0e}^{2}/4D$, and Θ is given as follows:

(3) $$\Theta =\frac{P_{e}\alpha l}{\lambda_{p}\kappa }\frac{\mathrm{dn}}{\mathrm{dT}}$$

where P_e_ is the excitation power at the sample (α), while dn and dT are the sample’s absorption coefficient and the temperature coefficient of the refractive index, respectively. The parameters κ and D are related through ρ and Cp by the following equation:

(4) $$\kappa =\rho c_{p}.D$$

The factor $m={\left(\omega_{p}/\omega_{e}\right)}^{2}$ is the mode-mismatching between the radii of excitation and the probe beams, and ν(z) is the geometrical factor defined and expressed as follows:

(5) $$\nu (z)=\frac{{z-a}_{p}}{z_{p}}+\frac{z_{p}}{L-z}\left[1+\frac{{\left({z-a}_{p}\right)}^{2}}{z_{p}^{2}}\right]$$

where *z* is the position of the sample in reference to the excitation beam waist, L is the distance from the sample to the detector, and a_p_, z_p_, and λ_p_ are the waist positions, Rayleigh parameter, and wavelength of the probe beam, respectively.

For TLS measurement, it is necessary to measure the experimental TLS signal using Equation (1), which has to be fitted as a function of time using Equation (2). In the process, Θ and D are taken as fitting parameters. The fitted value of D and the measured c can be used for calculating the κ of the sample using Equation (4). Three measurements were performed in each polyolefin homopolymer in order to determine the standard error. 

### 2.3. Characterization of Samples

#### 2.3.1. Molecular Characterization

The high-temperature gel permeation chromatography (HT-GPC) measurements were carried out at 150 °C with an HT-GPC fully integrated dual detector (PL-GPC 220, Agilent, Santa Clara, CA, USA) including differential refractive index (DRI) and bridge viscometry (BV 400) detectors from Agilent Technology Co. Three PLgel 10 μm MIXED-B columns (300 × 7.5 mm, Agilent), along with a guard column (50 × 7.5 mm, Agilent, Santa Clara, CA, USA), were used. 1,2,4-Trichlorobenzene (TCB) containing butylated hydroxytoluene (BHT) as a stabilizer was used as the eluent, at a flow rate of 1.0 mL/min (150 °C). The chromatograms were worked up from the RI signal, utilizing a narrow standard polystyrene calibration (11 points, ranging from 580 to 6,570,000 g/mol).

The polymers were dissolved by gentle shaking in TCB containing BHT (250 ppm) at a concentration of 1−1.2 mg/mL for 3 h at 160 °C, using an Agilent PL-SP 260VS sample preparation system. The samples were then filtered through a 2 μm stainless steel filter into the 2 mL glass GPC vials and run in the instrument with an injection volume (loop volume) of 200 µL at 150 °C, using TCB containing BHT (250 ppm) as an eluent. Data processing was performed using Cirrus GPC multi-detector software.

#### 2.3.2. Rheological Characterization

The rheological properties of the polymer samples were measured using a rheometric mechanical spectrometer equipped with an RMS 302e convection temperature device (CTD) (Anton Paar, Ostfildern, Germany). 

Oscillatory frequency sweeps ranging from 0.1 to 100 rad/s with a fixed strain were performed in N_2_ at 160 °C for HD, LD, and LLD, and at 190 °C for PP, to investigate the linear viscoelastic behavior of the samples in a frequency range of 0.01–625 rad/s with the strain amplitude of 1%, which was previously determined by strain sweep testing at a frequency of 10 rad/s at 160 °C for polyethylene and 190 °C for PP. Tests were carried out in parallel-plate geometry on disks with a thickness and diameter of 1 and 25 mm, respectively. Continuous and stable nitrogen purging of the environment chamber was necessary to inhibit oxidative degradation of the polymers. After the sample loading, an approximate 5 min equilibrium time was applied prior to each frequency sweep. All rheological data were collected using the RheoCompass software package (version number 2.43; 1.14.494 Ostfildern/Germany).

#### 2.3.3. Density

Density measurements were carried out on the basis of Archimedes’ principle, using the density determination kit designed for the AG204 Mettler-Toledo balance. The density of samples was determined by accurately (±0.01 mg) determining the weight of a sample specimen in the range of 10–20 g in air, and comparing it with its weight when immersed in butyl acetate (≥99%, Merck Co., Rahway, NJ, USA) at 23 °C.

#### 2.3.4. Thermal Properties

Calorimetry is the main technique for the determination of the thermal properties of polymers, and is the only method for direct measurement of the enthalpy associated with the thermal processes. We used the DSC method to directly measure some parameters—such as melting point, the heat of fusion, degree of crystallinity, and specific heat capacity (Cp)—of the polymeric samples.

The thermal properties of the samples were investigated by differential scanning calorimetry (DSC). The thermograms were recorded with a METTLER TOLEDO DSC822e instrument (Zurich, Switzerland) at a heating rate of 10 K/min under a N_2_ atmosphere. The DSC instrument was previously calibrated with indium and zinc under a dry nitrogen purge of 50 mL/min. Characteristic thermal properties such as degree of crystallinity, melting point, heat of fusion, and Cp were studied by means of STARe software. A standard 40 µL aluminum crucible with a pin and a pierced lid was used. The weights of the samples were approximately 7.9–9 mg. The experiments were performed by temperature programming in three segments as follows: (1) heating from 25 to 180 °C, (2) cooling from 180 to 25 °C, and (3) heating from 25 to 180 °C, all at 10 °C/min. The degree of crystallinity of samples was calculated from the DSC curve by dividing the measured heat of fusion by the heat of fusion of a 100% crystalline-related polymer (293.6 J/g for HD, LD, L and LD polyethylenes, LLD polyethylenes and 207.1 for PP).

The Cp of polymer samples was determined by the DSC method in the range of 10 to 40 °C in comparison to a sapphire disk, and with automatic blank curve subtraction. Temperature programming for Cp determination was as follows: segment (1) 10 °C for 4 min, segment (2) 10 to 40 °C at a rate of 20 °C/min, and segment (3) 40 °C for 2 min. The sample was heated from room temperature to 150 °C, cooled to −10 °C, and finally heated again to 150 °C, at a heating/cooling rate of 10 °C/min. The second heating curve was used to determine the heat of fusion, melting temperature (T_m_), and degree of crystallinity.

## 3. Results

### 3.1. Thermal Diffusivity 

**Figure 2:**
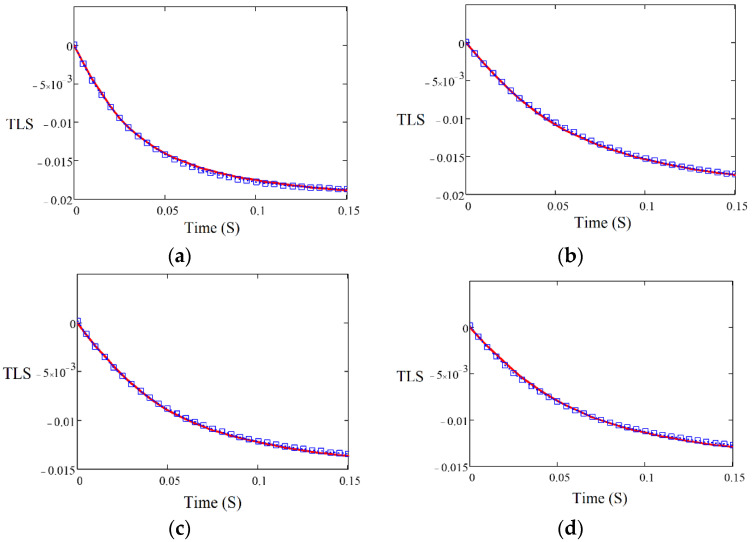
TLS signals for (**a**) HDPE, (**b**) LLDPE, (**c**) LDPE, and (**d**) PP as a function of time.

**Figure 3:**
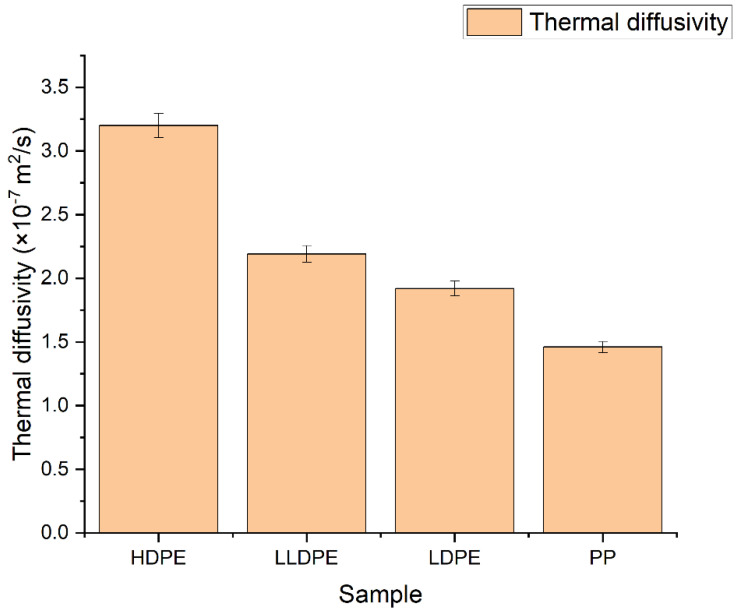
Thermal diffusivity of polyolefin homopolymers.

The D of four polyolefins was measured by the TLS technique, using the experimental setup shown in Figure 1, and the fitting was performed using Equation (2). Figure 2 shows the TLS signal (blue squares) fitted (red curve) for HDPE, LLDPE, LDPE, and PP using Equation (2) with the following parameters: $z_{p}=1000$ cm, $z_{p}=0.1$, cm, L = 100 cm, Θ = −0.014, and D = 3.20 × 10^−7^ m^2^/s, D = 2.19 × 10^−7^ m^2^/s, D = 1.92 × 10^−7^ m^2^/s, and D = 1.46 × 10^−7^ m^2^/s, respectively. The measured values of D for all samples are shown in Figure 3. There is a good agreement between the obtained values of D and those reported in the literature [24].

### 3.2. Thermal Conductivity

For calculating κ, first we used the Cp value of polymer samples, which was determined via automatic blank curve subtraction, compared with a sapphire disk (Figures S1–S4). It was found that in the series of polyethylenes, with increasing density, the value of Cp decreased (Table 2). The values of κ were compared with the literature measurements, and the analysis indicated that there was fairly good agreement [5].

### 3.3. Molecular Structure

**Figure 4:**
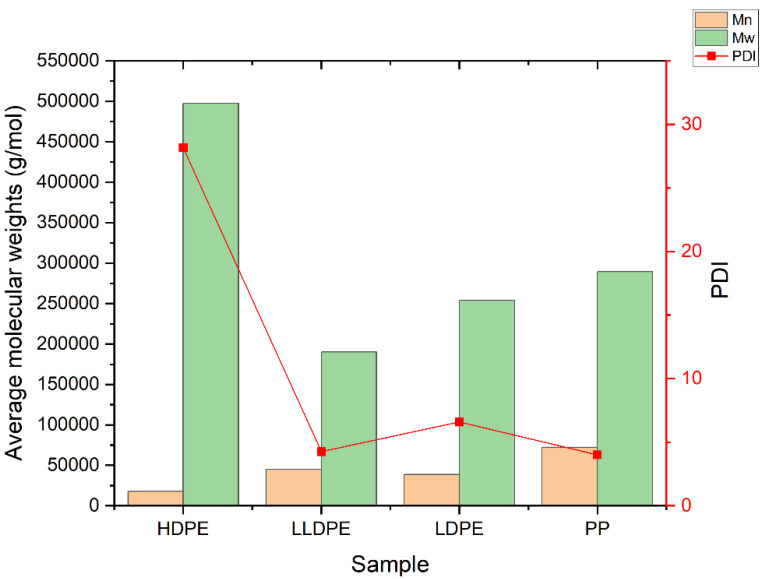
Molecular weights (M_n_, M_w_) and PDI of PP, LDPE, LLDPE, and HDPE.

**Figure 5:**
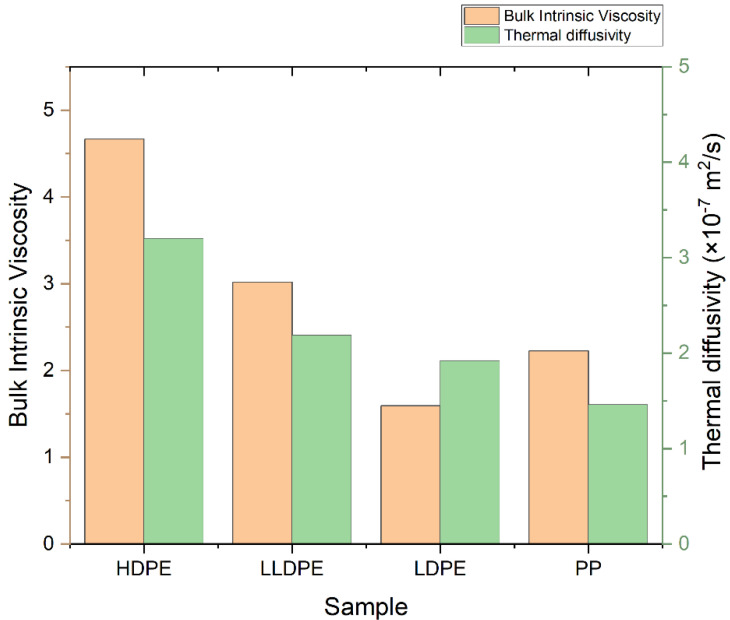
Bulk intrinsic viscosity and thermal diffusivity of PP, LDPE, LLDPE, and HDPE.

One of the most well-known methods for the characterization of polymers’ structures—especially polyolefins—is high-temperature gel permeation chromatography (HT-GPC). In addition to average molecular weights and molecular weight distribution (Table S1), which can be obtained by the GPC method, other information—such as the intrinsic viscosity, the amount of long-chain branches, and the radius of gyration—can also be obtained via this method. An HT-GPC fully integrated dual detector was used for the characterization of polymeric samples. Numerical data and diagrams of log M_w_ × IV vs. retention time, log M_w_ vs. retention, dw/dlogM vs. log Mw, Rg vs. log Mw, and IV and G^′^ vs. log Mw were obtained using this method (Table S1, Figures S9–S28), which provided good information about the microstructure of the samples. Figure 4 shows that LDPE, LLDPE, and PP have a narrower distribution (i.e., lower PDI) than HDPE, while HDPE has the lowest number average molecular weight (M_n_), but the highest weight average molecular weight (M_w_) and, thus, the highest polydispersity index in comparison to other samples, because HDPE is a bimodal resin produced by tandem processes that use a combination of two slurry reactor with a Ziegler–Natta catalyst. LDPE is prepared through a radical polymerization mechanism in a tubular reactor. In tubular reactors, due to the formation of fewer side chain branches, narrower polydispersity or lower PDI occurs relative to autoclave reactors. As shown in Figure 5, in the series of polyethylenes, we can see that the thermal diffusivity decreases with the decrease in intrinsic viscosity, so HDPE has higher intrinsic viscosity and thermal diffusivity.

#### 3.3.1. Branching Number

In polymers, one way to adjust the physical properties is to change the amount of branching. In polyolefins, there are two types of lateral branches in terms of branch length. If the side chain contains six or more carbons it is long-chain branching (LCB); otherwise, it is short-chain branching (SCB). Obviously, the effect of the long side branch on the thermal and mechanical properties of the polymer will be stronger. In the GPC method with a viscometry detector, in addition to average molecular weight distribution, long-chain branches are measured. Long-chain branches can affect several physical parameters, such as density, degree of crystallinity, melting point, viscosity, processability, and MFI.

**Figure 6:**
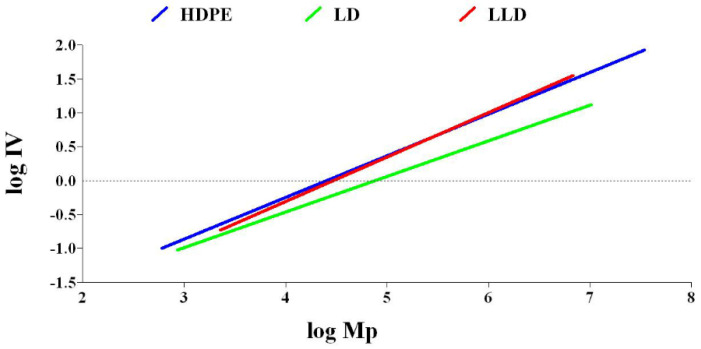
Diagram of log intrinsic viscosity vs. log molecular weight for polyethylenes.

**Figure 7:**
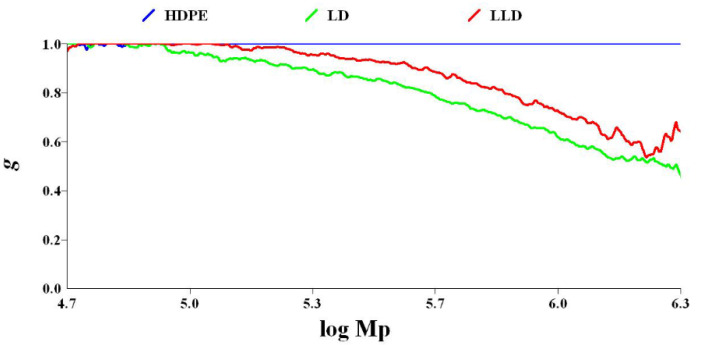
Diagram of g vs. log molecular weight for polyethylenes.

**Figure 8:**
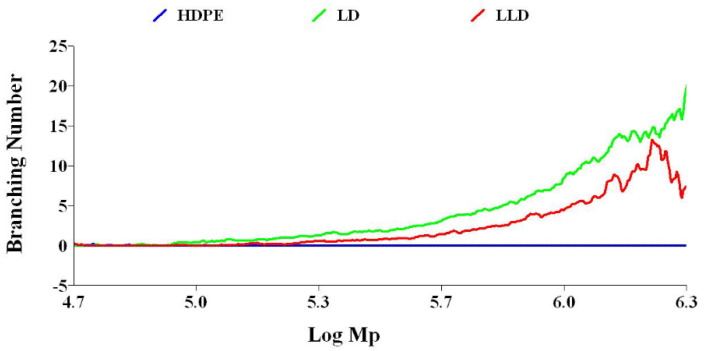
Diagram of branching number vs. log molecular weight for polyethylenes.

The LCB number of polyolefins can be determined by comparing the behavior of a branched polymer with that of a linear chain analog with the same chemistry. Compared to a linear polymer, the branched polymer will be more compact at any given molecular weight. As a result, the branched molecule has a smaller hydrodynamic volume radius of gyration as well as a lower intrinsic viscosity. The degree of branching in a material can be modeled based on the difference in intrinsic viscosity (dL/g) (as determined by GPC/Viscometry) or the radius of gyration (as determined by GPC/Light Scattering) of a branched sample compared to a linear analog. Plots of log intrinsic viscosity (Mark–Houwink plots) and log radius of gyration (conformation plots) versus log molecular weight of PE samples are illustrated in Figure 6 and Figure 7, respectively. The differences in the behavior of the two polymers can be used to calculate values of G^′^ (the intrinsic viscosity contraction factor), which can be then considered in various structural models to estimate the degree of branching. The degree of branching is normally expressed as a branching number (Bn), defined as the number of branches per 1000 carbons. Figure 8 shows the diagram of calculated G^′^ in terms of log molecular weight for PE samples. As expected, increasing the degree of branching directly reduces the polymer density. Although PP does not have long branches, there is a small branch of methyl on all of its monomers, making its density very low. Consequently, κ decreases, as shown in Table 2.

The ratio between the intrinsic viscosity or radius of gyration of the branched polymer and the linear polymer can be used to calculate values of G^′^ (the intrinsic viscosity contraction factor) or g (the radius of gyration contraction factor), respectively, according to the following equations:

(6) $$g^{\prime }=\left(\frac{IV_{branched}}{IV_{linear}}\right)$$

(7) $$g=\left(\frac{Rg_{branched}}{Rg_{linear}}\right)$$

where $g^{\prime }=g^{\varepsilon }$, and ε (structure factor) = 0.5–1.5—usually 0.75. The value g can then be input into various structural models (number average ternary-branching model; see Equation (8)) to estimate the B_n_.

(8) g=[(1+Bn7)12+4Bn9π]−12

#### 3.3.2. Rheology

**Figure 9:**
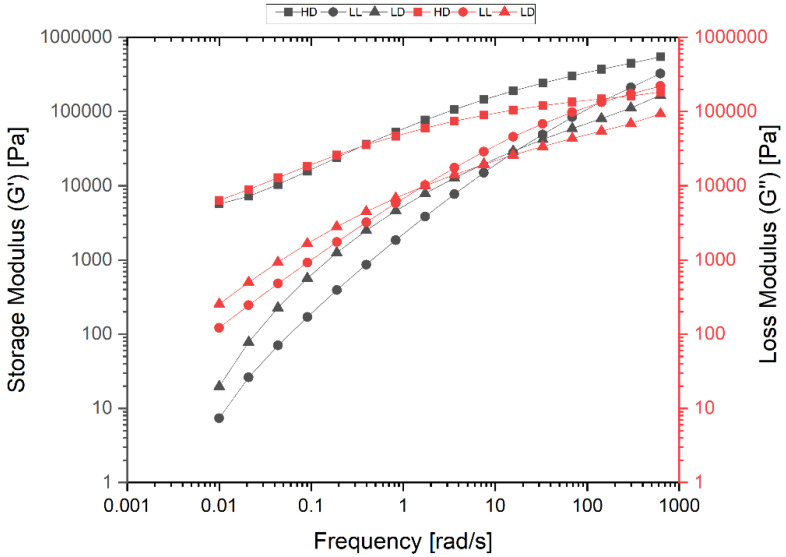
Diagram of storage modulus G^′^; and loss modulus G^″^ in terms of angular frequency ω for HDPE (HD), LLDPE (LL), and LDPE (LD).

In order to completely characterize the samples, the rheology of the samples was investigated by oscillatory frequency sweep tests. Figure 9 shows the diagrams of storage modulus G^′^ and loss modulus G^″^ in terms of angular frequency ω (rad/s). By increasing the angular frequencies, the crossover point (COP) between the G^′^ and G^″^ curves indicates a transition from a more viscous-like deformation behavior to a more elastic-like deformation behavior. Therefore, the COP is a criterion for qualitative product characterization. One of the most important pieces of information we can get from the position of the COP is a qualitative measurement of a polymer’s average molecular weight.

The shift of the COP to higher angular frequencies indicates a lower average molecular weight, while the shift of the COP to lower angular frequencies indicates a higher average molecular weight, because at higher angular frequencies shorter molecules remain mobile, whereas longer molecules become immobile at lower angular frequencies

The diagrams show that the average molar mass of the polymers increased with the shift of the COP to lower angular frequencies (ω_COP_). Therefore, HDPE (ω_COP_ = 0.39 s^−1^) has a higher average molar weight than LDPE, with ω_COP_ = 7.50 s^−1^. LLDPE with ω_COP_ = 140 s^−1^ is much lower than LDPE. These relative values are confirmed by the quantitative information obtained via the GPC method for average molecular weight M_w_ (Table S1).

#### 3.3.3. Crystallinity

Among all of the factors affecting κ and D, the percentage of crystallinity is the most important factor, and is affected by the type, number, and length of side branches of the main chain. Most polyolefins have crystalline and amorphous parts, which are also different in different grades. In the crystalline part of the polymers, atoms are regularly arranged in a very close manner, so they can effectively vibrate in their equilibrium position. Therefore, heat is transferred quickly across the direction of the molecular chain, so with increasing crystallinity percentage, in addition to mechanical properties, processing properties, melt flow properties, and thermal properties (such as κ) also change. Increasing the number and length of short-chain branches or long-chain branches reduces the amount of polymer crystal areas, because the side branches do not allow the main chains to approach one another and fit into a crystalline structure.

**Figure 10:**
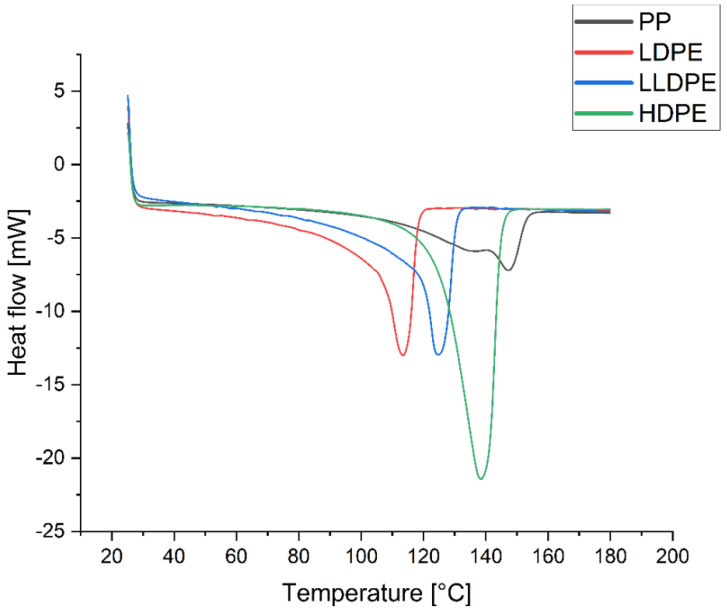
Thermograms of PP, LDPE, LLDPE, and HDPE.

**Figure 11:**
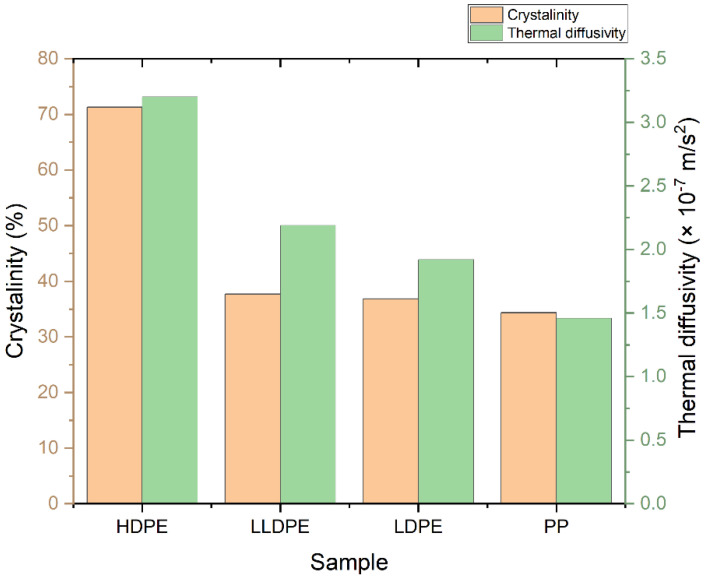
The percentage crystallinity and D of polyolefins.

The degree of crystallinity of samples was measured by using the DSC curve (Figure 10 and Figures S5–S8), dividing the measured heat of fusion of each sample by the heat of fusion of a 100% crystalline-related polymer (293.6 J/g for polyethylenes and 207.1 for PP). As expected, the results obtained from the thermograms show that the order of degree of crystallinity is as follows: HDPE > LLDPE > LDPE > PP; they also indicate that the value of D increases with the crystallinity of the PE samples (Figure 11).

## 4. Conclusions

In this work, the D and κ of widely used polyolefin homopolymers such as HDPE, LDPE, LLDPE, and PP were investigated using the TLS technique. We found a correlation between D, κ, and the degree of crystallinity, long-chain branching number, and thermal, molecular, and rheological properties of the polymers. The values of D and k obtained are in good agreement with those that have been reported in the literature. The measurement of D by the TL technique is simple, accurate, and non-destructive, where the required time and quantity of the sample are minimal compared with other conventional methods. It is worth mentioning that the TLS technique demonstrated the capability to measure the thermal properties of very thin (100 µm thickness) polymeric samples. Additionally, the results showed that among the molecular properties, the long-chain branching number can affect the crystallinity of PE, which can directly affect the D and κ of PE. This artifact can be used for designing polymeric materials with desired values of D and κ, which is crucial for industrial applications. The results demonstrated good repeatability and accuracy, comparable with other techniques; however, TLS is an alternative and highly sensitive method able to measure very thin materials with very low absorbance.

## Figures and Tables

**Table 1:** Mass flow index of commercial grade polyolefins.

Sample	Grade	MFI (g/10 min)
HDPE	DR-720	0.2 ± 0.02 ^1^
LLDPE	22B02	2 ± 0.02 ^1^
LDPE	2420E02	1.9 ± 0.02 ^1^
PP	ZR340R	25 ± 3 ^2^

^1^: At 190 ˚C; ^2^: at 230 ˚C ASTM D1238.

**Table 2:** D, κ, ρ, and Cp of HDPE, LLDPE, LDPE, and PP.

Sample	D(×10^−7^), m^2^/s	ρ, g/cm^3^	Cp ^1^*,* J/gK	κ, W/mK
HDPE	3.20	0.958	1.76	0.54
LLDPE	2.19	0.922	2.03	0.41
LDPE	1.92	0.921	2.09	0.37
PP	1.46	0.903	1.67	0.22

^1^ Cp measured at 25 °C.

## Data Availability

The data presented in this study are available upon request from the corresponding author.

## Supplementary Materials

The following supporting information can be downloaded at: https://www.mdpi.com/article/10.3390/polym14132707/s1, Figure S1: Thermogram for calculation of Cp of HDPE; Figure S2: Thermogram for calculation of Cp of LLDPE; Figure S3: Thermogram for calculation of Cp of LDPE; Figure S4: Thermogram for calculation of Cp of PP; Figure S5: DSC Thermogram for calculation of crystallinity of PP; Figure S6: DSC Thermogram for calculation of crystallinity of HDPE; Figure S7: DSC Thermogram for calculation of crystallinity of LLDPE; Figure S8: DSC Thermogram for calculation of crystallinity of LDPE; Figure S9: GPC chromatogram of HDPE, Figure S10: Diagram of log Mw vs Retention Time for HDPE; Figure S11: Diagram of dw/dlogM vs log Mw for HDPE, Figure S12: Diagram of Rg vs log Mw for HDPE; Figure S13: Diagram of IV and G^′^ vs log Mw for HDPE; Figure S 14: GPC chromatogram of LLDPE, Figure S15: Diagram of log Mw vs Retention Time for LLDPE, Figure S16: Diagram of dw/dlogM vs log Mw for LLDPE, Figure S17: Diagram of Rg vs log Mw for LLDPE, Figure S18: Diagram of IV and G^′^ vs log Mw for LLDPE; Figure S19: GPC chromatogram of LDPE; Figure S20: Diagram of log Mw vs Retention Time for LDPE, Figure S21: Diagram of dw/dlogM vs log Mw for LDPE, Figure S22: Diagram of IV and G^′^ vs log Mw for LDPE; Figure S23: Diagram of Rg vs log Mw for LDPE, Figure S24: GPC chromatogram of PP; Figure S25: Diagram of log Mw vs Retention Time for PP; Figure S26: Diagram of dw/dlogM vs log Mw for PP, Figure S27: Diagram of IV and G^′^ vs log Mw for PP, Figure S28: Diagram of Rg vs log Mw for PP; Table S1: Average molecular weight, PDI, weight fraction, and bulk intrinsic viscosity of samples obtained by GPC analysis.

## References

[1] Palacios A., Cong L., Navarro M., Ding Y., Barreneche C. (2019). Thermal conductivity measurement techniques for characterizing thermal energy storage materials—A review. Renew. Sustain. Energy Rev..

[2] Guo Y., Ruan K., Shi X., Yang X., Gu J. (2020). Factors affecting thermal conductivities of the polymers and polymer composites: A review. Compos. Sci. Technol..

[3] Weingrill H., Hohenauer W., Resch-Fauster K., Zauner C. (2019). Analyzing Thermal Conductivity of Polyethylene-Based Compounds Filled with Copper. Macromol. Mater. Eng..

[4] Hosier I.L., Andritsch T., Vaughan A.S., Stevens G.C., McAllister N., Basu S., German I. (2021). Enhanced Boron Nitride/Polyolefin Blends for High Voltage Applications. IEEE Trans. Nanotechnol..

[5] Chaudhry A., Mabrouk A., Abdala A. (2020). Thermally enhanced pristine polyolefins: Fundamentals, progress and prospective. J. Mater. Res. Technol..

[6] Leite N., Cella N., Vargas H., Miranda L. (1987). Photoacoustic measurement of thermal diffusivity of polymer foils. J. Appl. Phys..

[7] Zhang X., Fujii M. (2003). Measurements of the thermal conductivity and thermal diffusivity of polymers. Polym. Eng. Sci..

[8] dos Santos W.N., Mummery P., Wallwork A. (2005). Thermal diffusivity of polymers by the laser flash technique. Polym. Test..

[9] Subhani S., Ghali V. (2019). Measurement of thermal diffusivity of fiber reinforced polymers using quadratic frequency modulated thermal wave imaging. Infrared Phys. Technol..

[10] Venerus D., Schieber J., Iddir H., Guzman J., Broerman A. (1999). Measurement of thermal diffusivity in polymer melts using forced Rayleigh light scattering. J. Polym. Sci. Part B Polym. Phys..

[11] dos Santos W.N., dos Santos J.N., Mummery P., Wallwork A. (2010). Thermal diffusivity of polymers by modified angström method. Polym. Test..

[12] Boué C., Holé S. (2012). Infrared thermography protocol for simple measurements of thermal diffusivity and conductivity. Infrared Phys. Technol..

[13] Lang S. (1989). Technique for the measurement of thermal diffusivity based on the Laser Intensity Modulation Method (LIMM). Ferroelectrics.

[14] Hashimoto T., Matsui Y., Hagihara A., Miyamoto A. (1990). Thermal diffusivity measurement of polymer films by the temperature wave method using joule-heating. Thermochim. Acta.

[15] Choy C., Yang G., Wong Y. (1997). Thermal diffusivity of polymer films by pulsed photothermal radiometry. J. Polym. Sci. Part B Polym. Phys..

[16] Wong P., Fung P., Tam H. (1998). Low thermal diffusivity measurements of thin films using mirage technique. J. Appl. Phys..

[17] Bauer S., De Reggi A.S. (1996). Pulsed electrothermal technique for measuring the thermal diffusivity of dielectric films on conducting substrates. J. Appl. Phys..

[18] Cabrera H., Mendoza D., Benítez J., Flores C.B., Alvarado S., Marín E. (2015). Thermal diffusivity of few-layers graphene measured by an all-optical method. J. Phys. D Appl. Phys..

[19] Cabrera H., Matroodi F., Cabrera-Díaz H.D., Ramírez-Miquet E.E. (2020). Frequency-resolved photothermal lens: An alternative approach for thermal diffusivity measurements in weak absorbing thin samples. Int. J. Heat Mass Transf..

[20] Hansen D., Ho C.C. (1965). Thermal conductivity of high polymers. J. Polym. Sci. Part A Gen. Pap..

[21] Washo B.D., Hansen D. (1969). Heat conduction in linear amorphous high polymers: Orientation anisotropy. J. Appl. Phys..

[22] Bai L., Zhao X., Bao R.-Y., Liu Z.-Y., Yang M.-B., Yang W. (2018). Effect of temperature, crystallinity and molecular chain orientation on the thermal conductivity of polymers: A case study of PLLA. J. Mater. Sci..

[23] Marcano A., Cabrera H., Guerra M., Cruz R.A., Jacinto C., Catunda T. (2006). Optimizing and calibrating a mode-mismatched thermal lens experiment for low absorption measurement. JOSA B.

[24] Bandrup J., Immergut E.H. (1989). Polymer Handbook.

