# Supplementary material for: Thermal Diffusivity and Conductivity of Polyolefins by Thermal Lens Technique
Source: Polymers (Basel). 2022 Jul 1;14(13):2707. doi: 10.3390/polym14132707 (PMC9269369; doi:10.3390/polym14132707)
Supplement: Supplementary file 1 [file polymers-14-02707-s001.zip › polymers-1742239-supplementary.pdf]

Supporting information for

Thermal Diffusivity and Conductivity of Polyolefins by Thermal Lens Technique

Behnaz Abbasgholi-NA [1,2], Seyed Reza Nokhbeh [3], Osamah A. Aldaghri [4], Khalid Hassan Ibnaouf [4], Nawal Madkhali [4,*] and Humberto Cabrera [1*]

[1] Optics Lab, STI Unit, The Abdus Salam International Centre for Theoretical Physics, Trieste 34151, Italy
[2] NanoInnovation Laboratory, Elettra-Sincrotrone Trieste S.C.P.A, Trieste, Italy
[3] Department of Chemistry, Ferdowsi University of Mashhad, Mashhad, Iran
[4] Physics Department, College of Sciences, Imam Mohammad Ibn Saud Islamic University (IMSIU), Riyadh 13318, Saudi Arabia; odaghri@imamu.edu.sa (O.A.A.); khiahmed@imamu.edu.sa (K.H.I.)
* Correspondence: hcabrera@ictp.it (H.C.); namadkhali@imamu.edu.sa (N.M.)

Figures & Table		page
Fig. S1	Thermogram for calculation of Cp of HDPE	2
Fig. S2	Thermogram for calculation of Cp of LLDPE	3
Fig. S3	Thermogram for calculation of Cp of LDPE	3
Fig. S4	Thermogram for calculation of Cp of PP	4
Fig. S5	DSC Thermogram for calculation of crystallinity of PP	4
Fig. S6	DSC Thermogram for calculation of crystallinity of HDPE	5
Fig. S7	DSC Thermogram for calculation of crystallinity of LLDPE	5
Fig. S8	DSC Thermogram for calculation of crystallinity of LDPE	6
Fig. S9	GPC chromatogram of HDPE	6
Fig. S10	Diagram of log Mw vs Retention Time for HDPE	6
Fig. S11	Diagram of dw/dlogM vs log Mw for HDPE	7
Fig. S12	Diagram of Rg vs log Mw for HDPE	7
Fig. S13	Diagram of IV and g' vs log Mw for HDPE	7
Fig. S14	GPC chromatogram of LLDPE	8
Fig. S15	Diagram of log Mw vs Retention Time for LLDPE	8
Fig. S16	Diagram of dw/dlogM vs log Mw for LLDPE	8
Fig. S17	Diagram of Rg vs log Mw for LLDPE	9
Fig. S18	Diagram of IV and g' vs log Mw for LLDPE	9
Fig. S19	GPC chromatogram of LDPE	9
Fig. S20	Diagram of log Mw vs Retention Time for LDPE	10

Fig. S21	Diagram of dw/dlogM vs log Mw for LDPE	10
Fig. S22	Diagram of IV and g' vs log Mw for LDPE	10
Fig. S23	Diagram of Rg vs log Mw for LDPE	11
Fig. S24	GPC chromatogram of PP	11
Fig. S25	Diagram of log Mw vs Retention Time for PP	11
Fig. S26	Diagram of dw/dlogM vs log Mw for PP	12
Fig. S27	Diagram of IV and g' vs log Mw for PP	12
Fig. S28	Diagram of Rg vs log Mw for PP	12
Table S1	Average molecular weights, PDI, weight fraction, and bulk intrinsic viscosity of samples obtained by GPC analysis	13

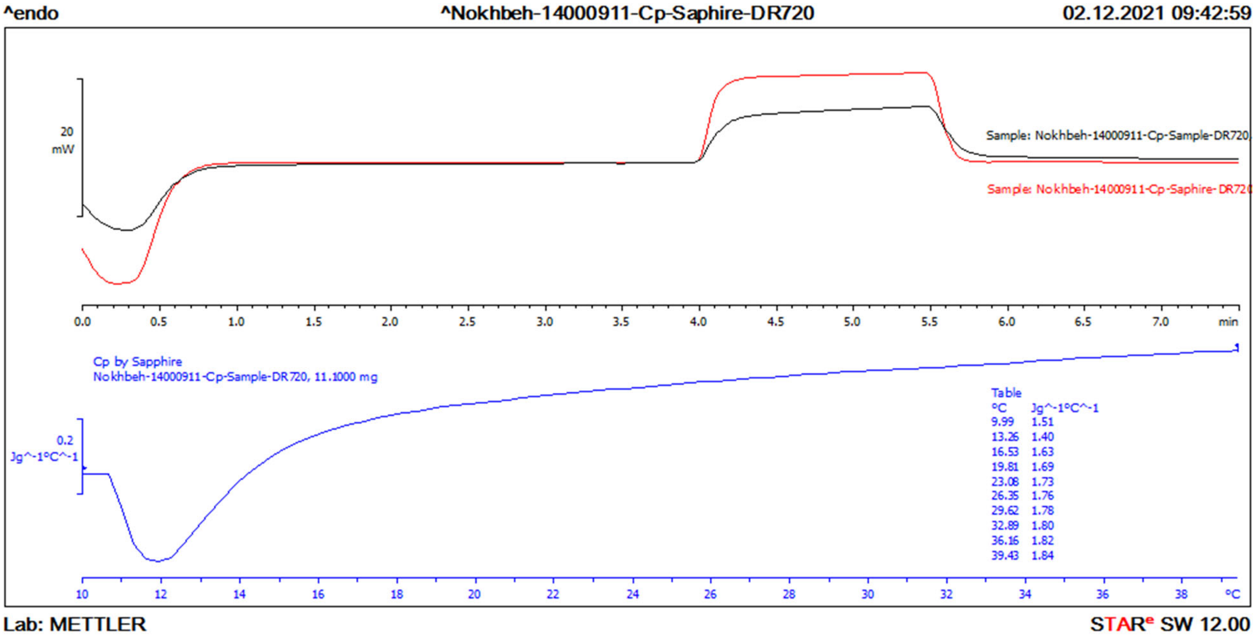

Figure S1: Thermogram for calculation of Cp of HDPE

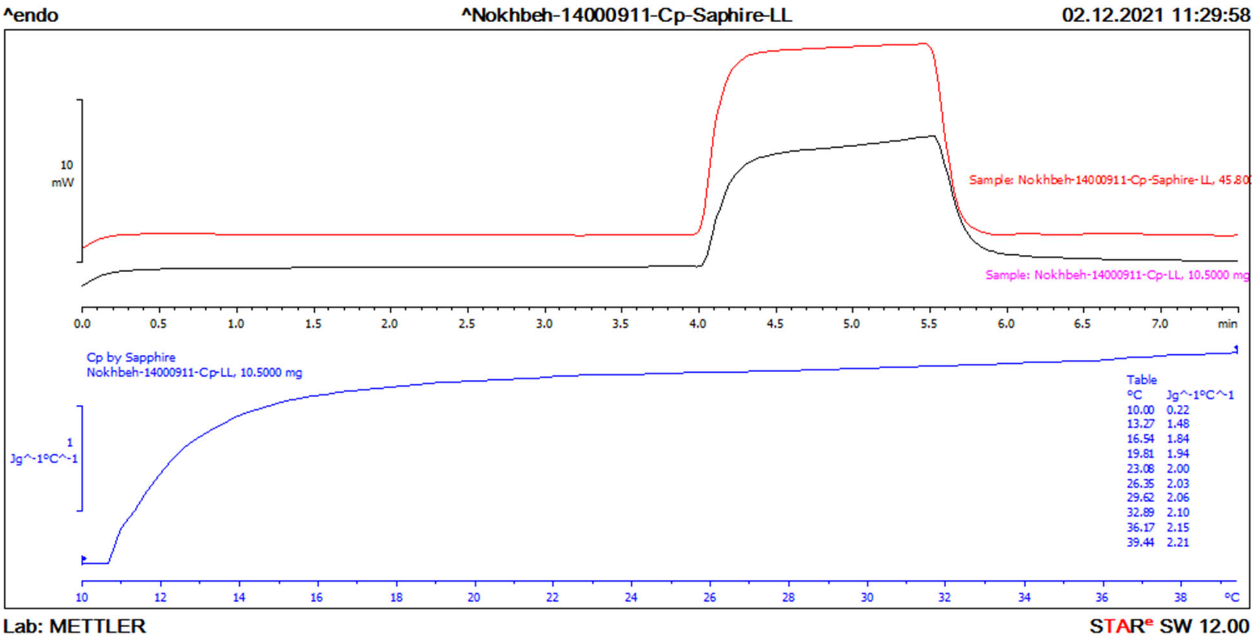

Figure S2: Thermogram for calculation of Cp of LLDPE

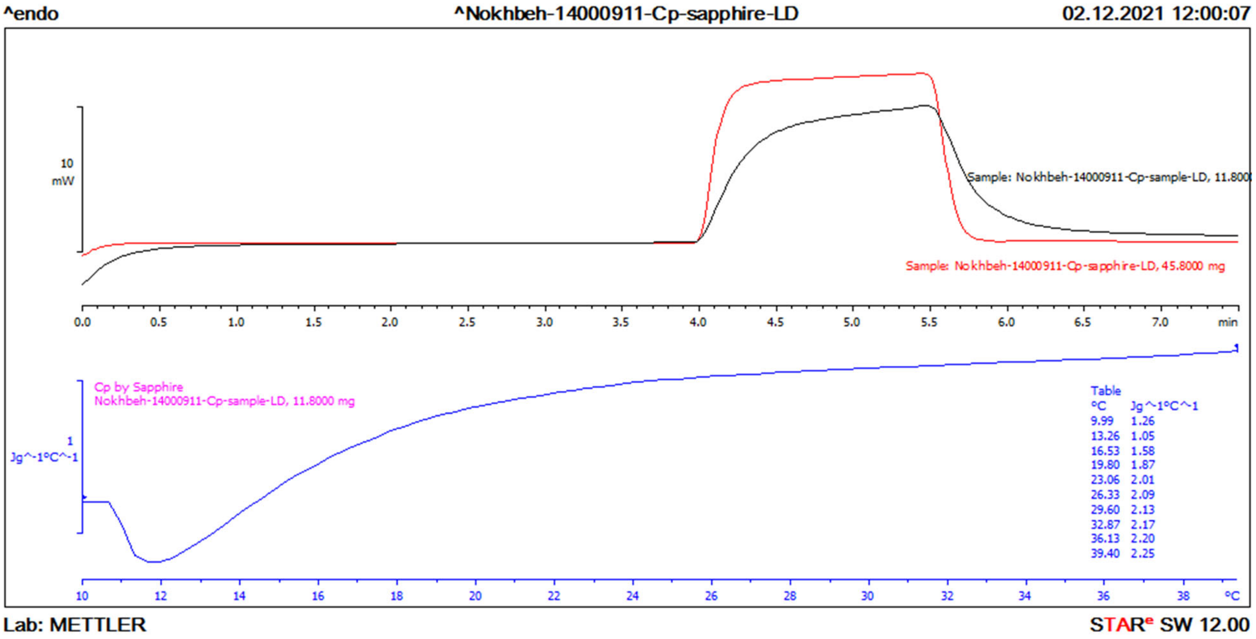

Figure S3: Thermogram for calculation of Cp of LDPE

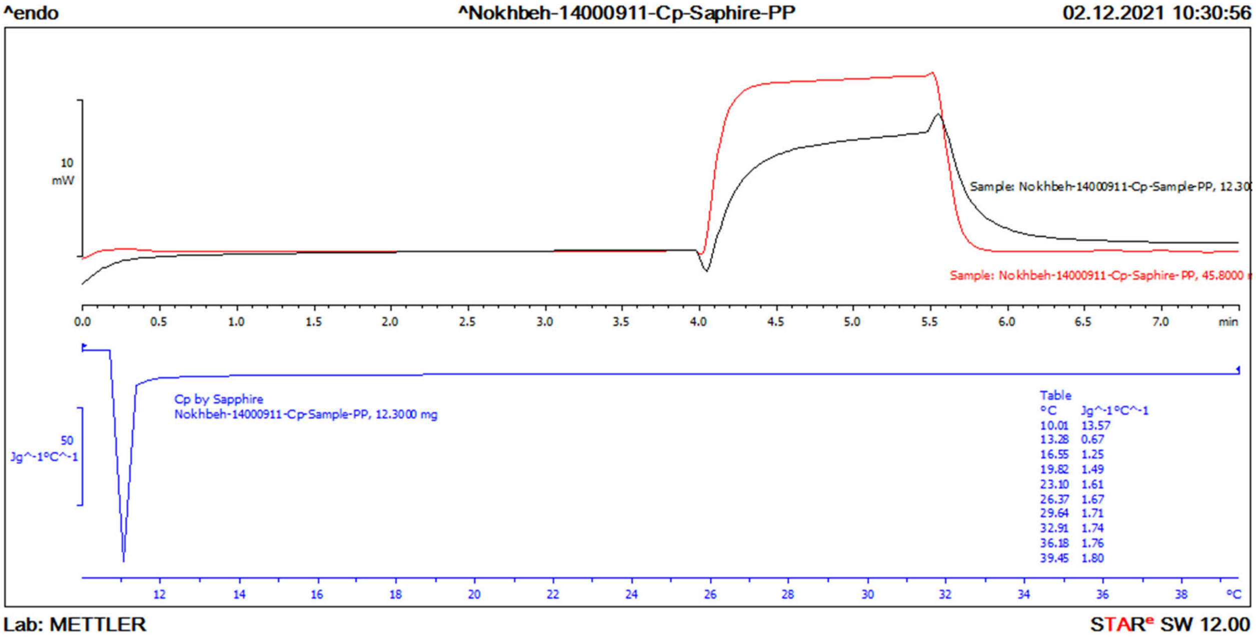

Figure S4: Thermogram for calculation of Cp of PP

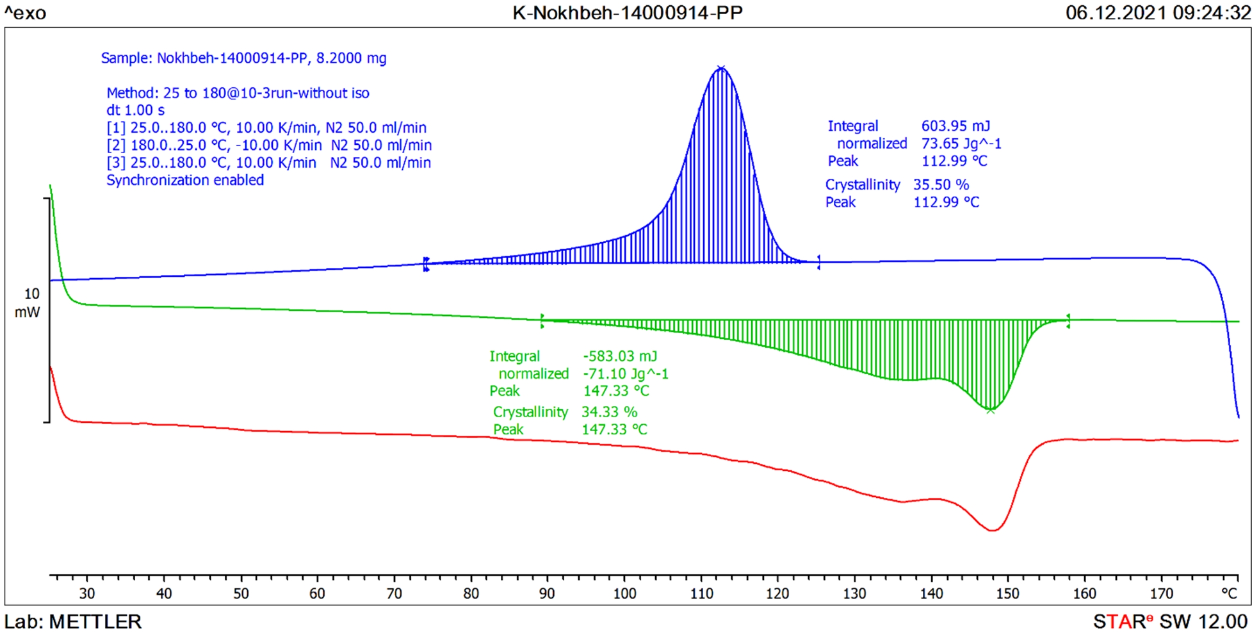

Figure S5: DSC Thermogram for calculation of crystallinity of PP

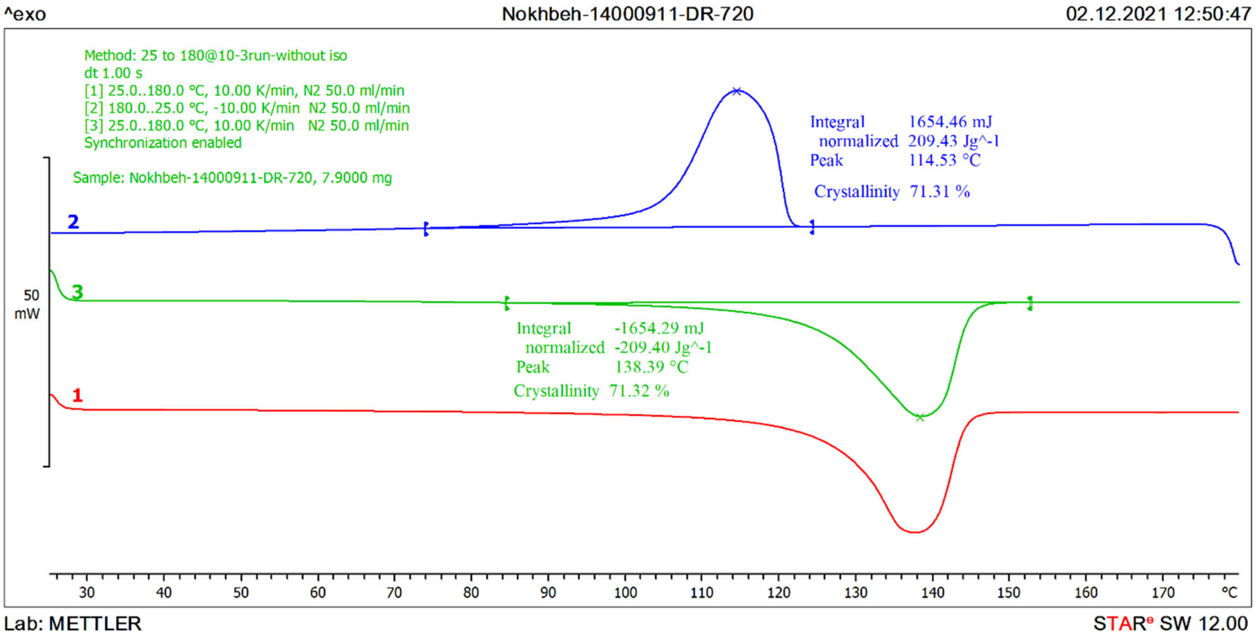

Figure S6: DSC Thermogram for calculation of crystallinity of HDPE

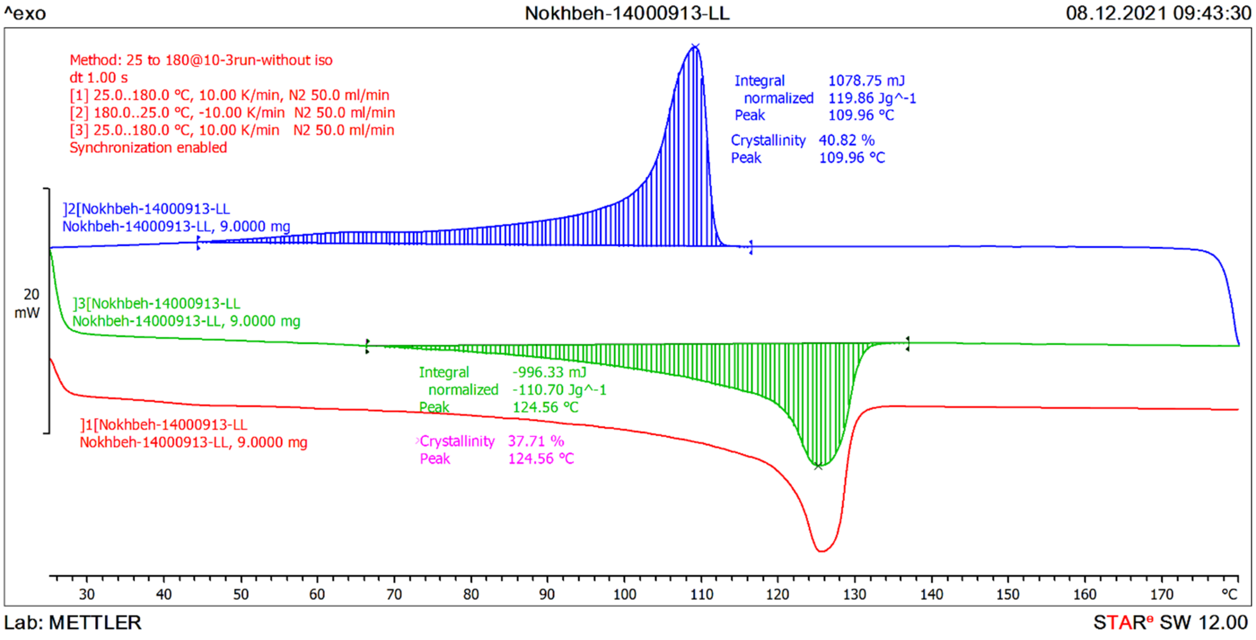

Figure S7: DSC Thermogram for calculation of crystallinity of LLDPE

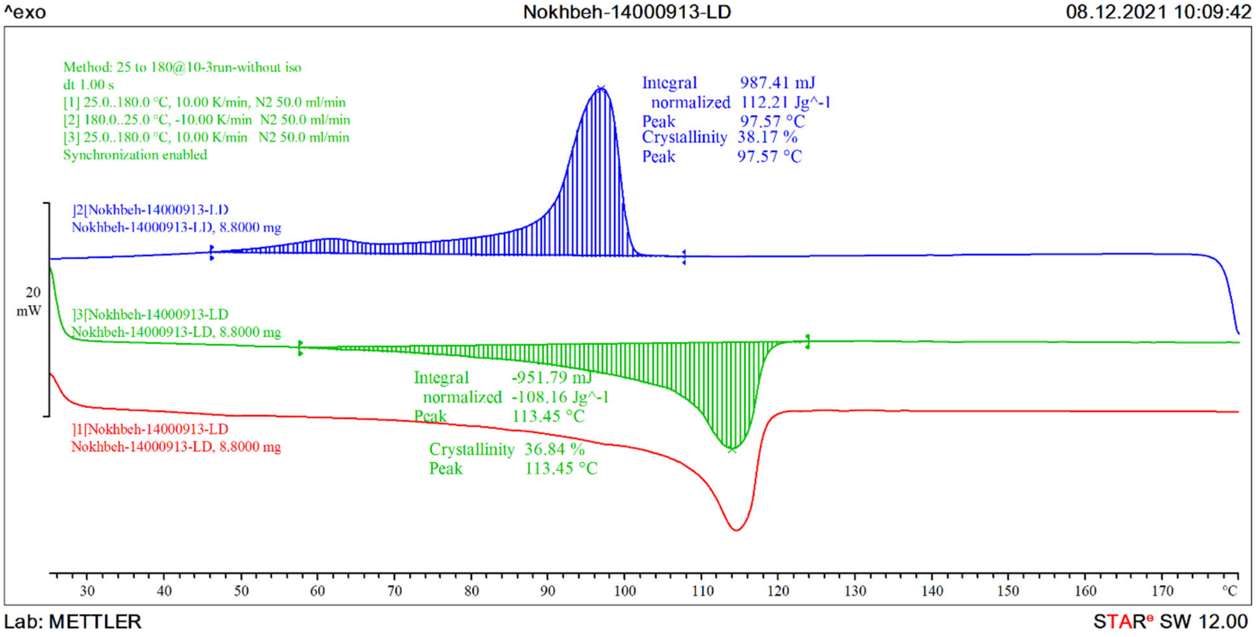

Figure S8: DSC Thermogram for calculation of crystallinity of LDPE

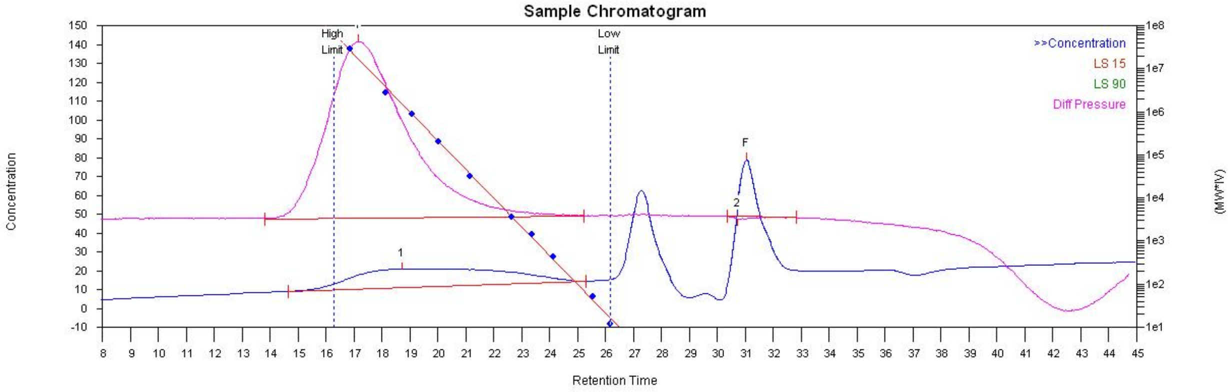

Figure S9: GPC chromatogram of HDPE

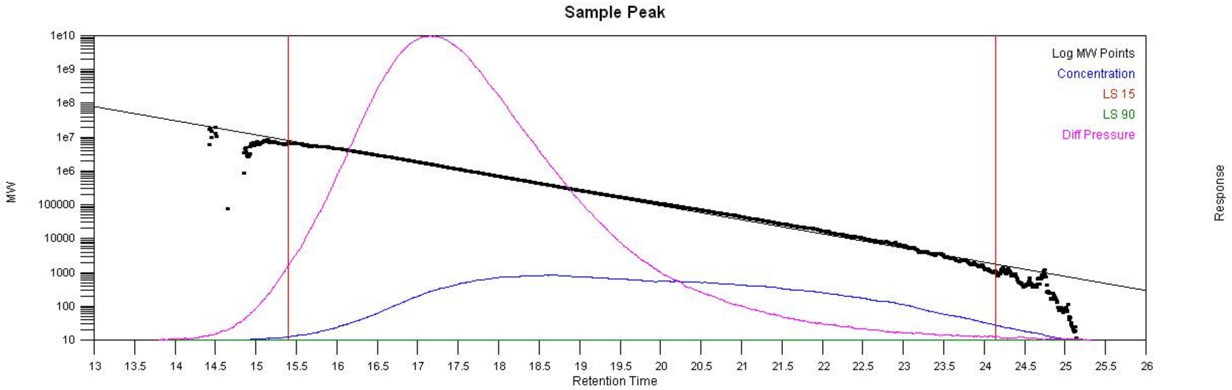

Figure S10: Diagram of log Mw vs Retention Time for HDPE

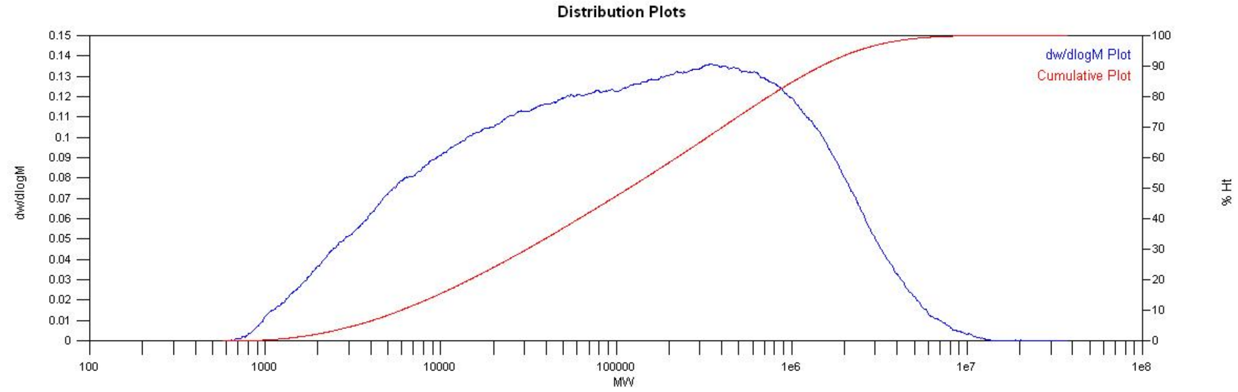

Figure S11: Diagram of dw/dlogM vs log Mw for HDPE

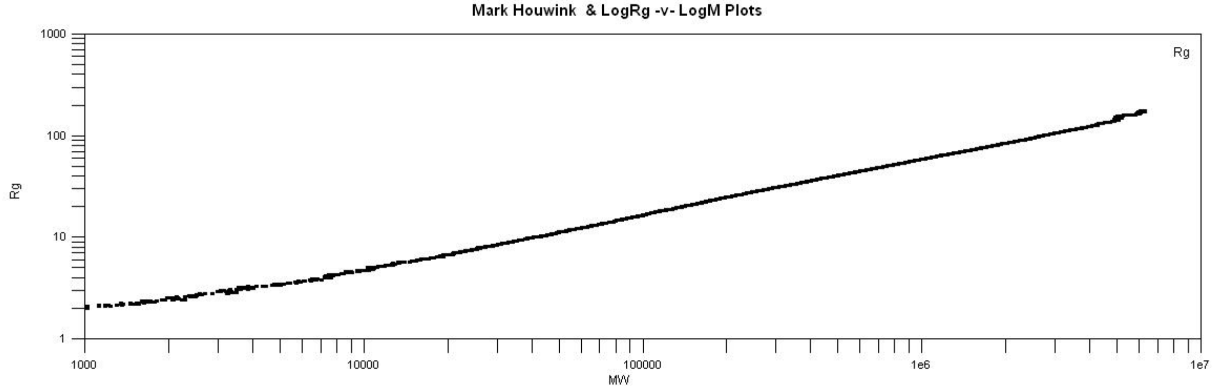

Figure S12: Diagram of Rg vs log Mw for HDPE

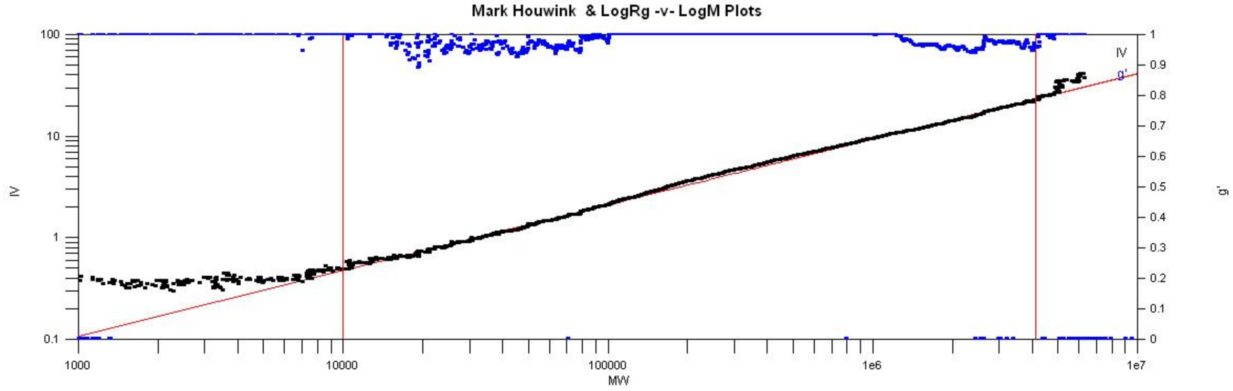

Figure S13: Diagram of IV and g' vs log Mw for HDPE

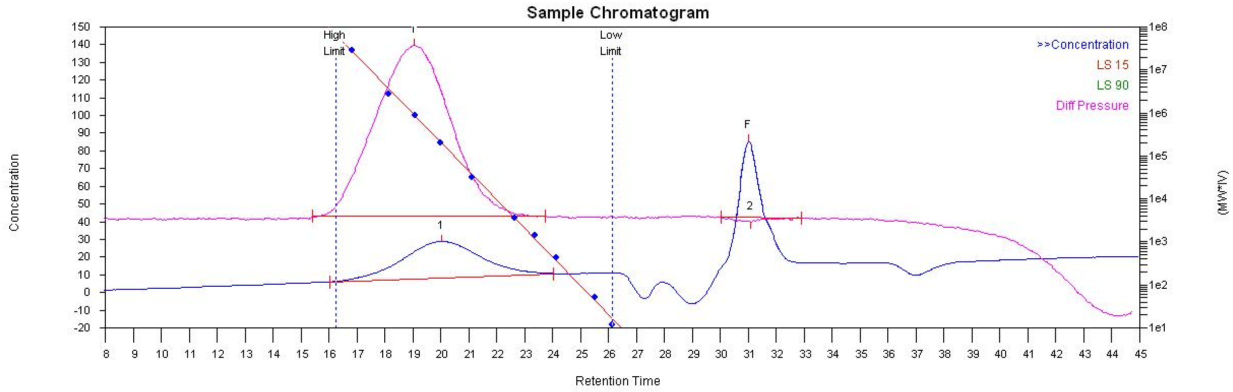

Figure S14: GPC chromatogram of LLDPE

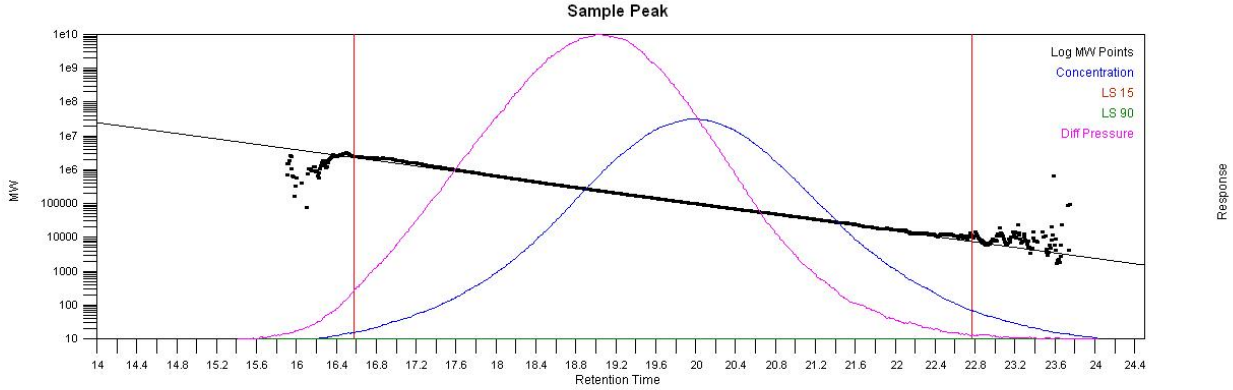

Figure S15: Diagram of log Mw vs Retention Time for LLDPE

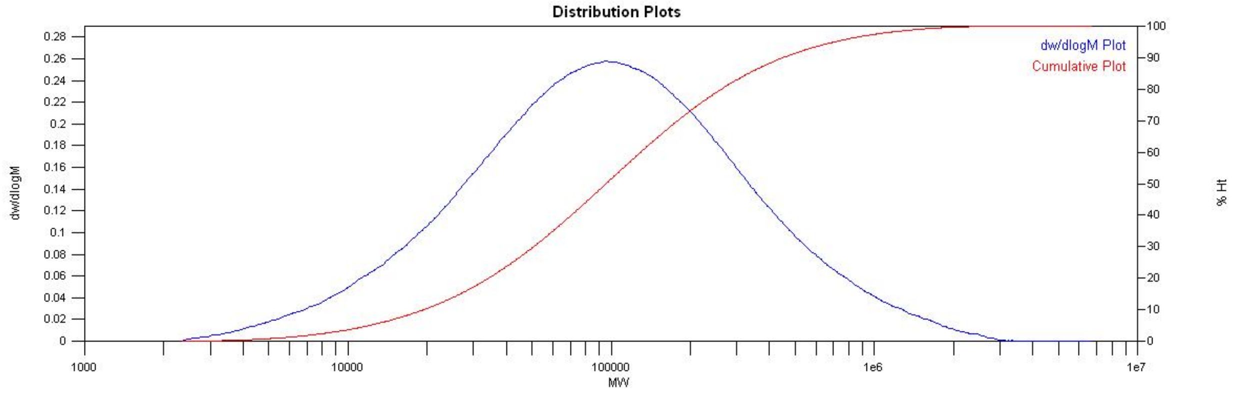

Figure S16: Diagram of dw/dlogM vs log Mw for LLDPE

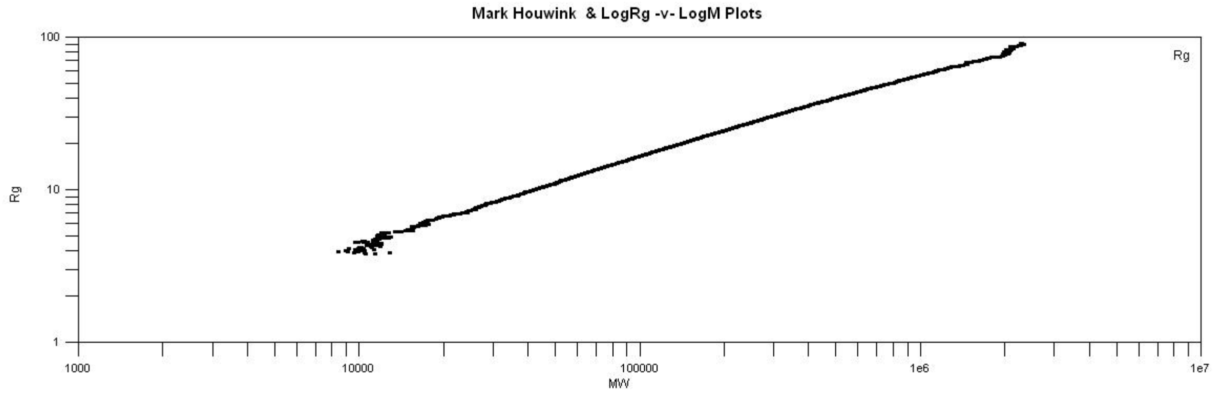

Figure S17: Diagram of Rg vs log Mw for LLDPE

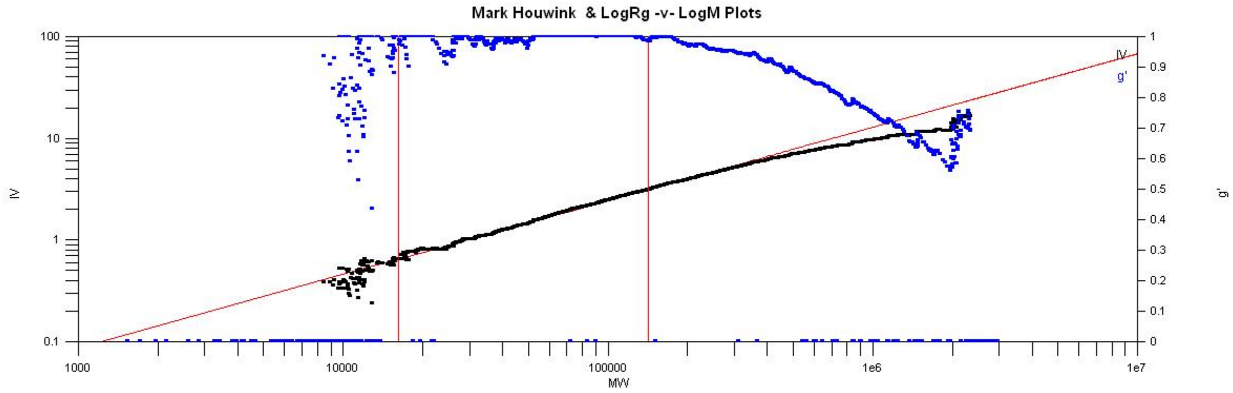

Figure S18: Diagram of IV and g' vs log Mw for LLDPE

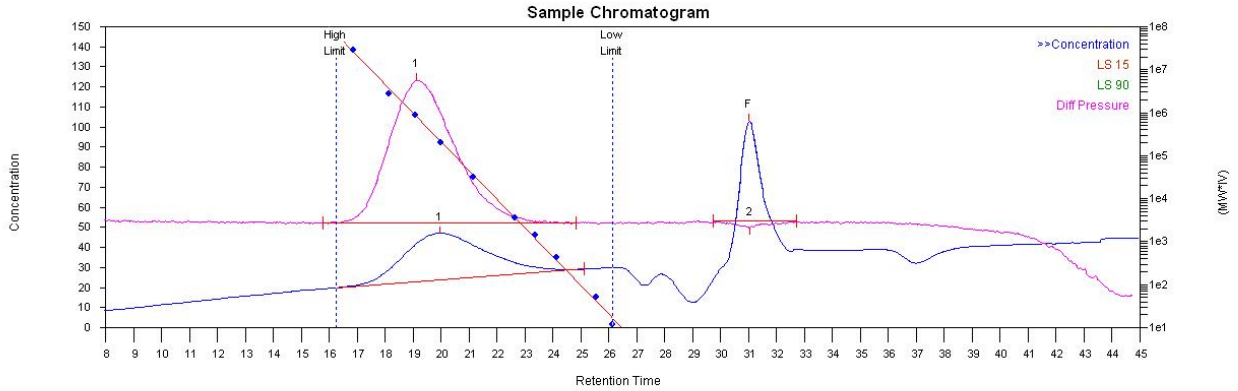

Figure S19: GPC chromatogram of LDPE

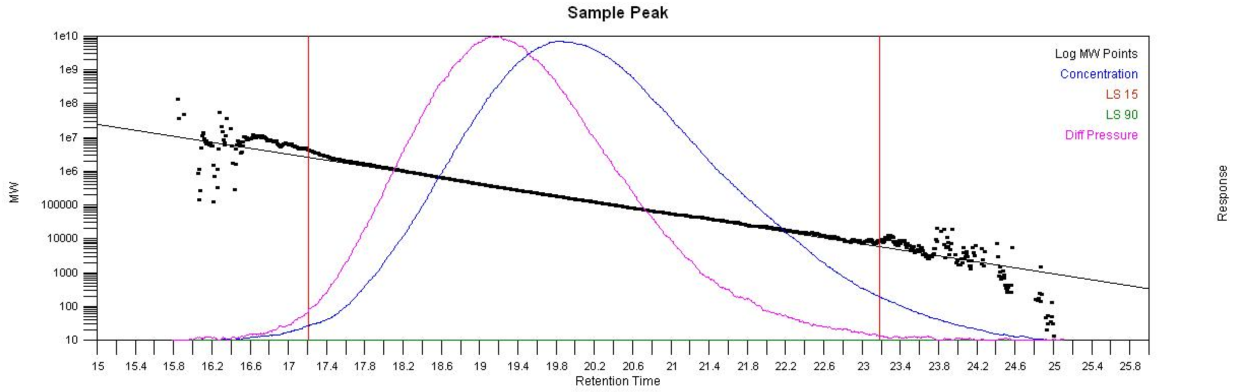

Figure S20: Diagram of log Mw vs Retention Time for LDPE

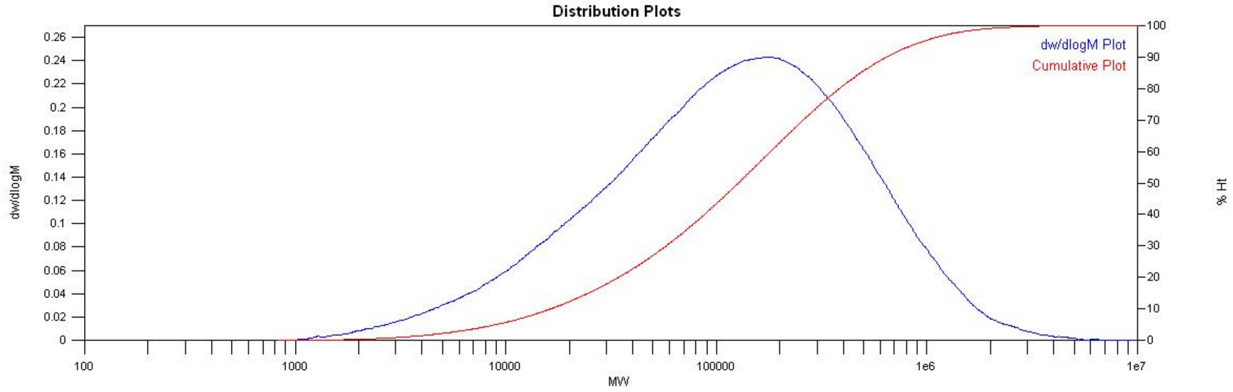

Figure S21: Diagram of dw/dlogM vs log Mw for LDPE

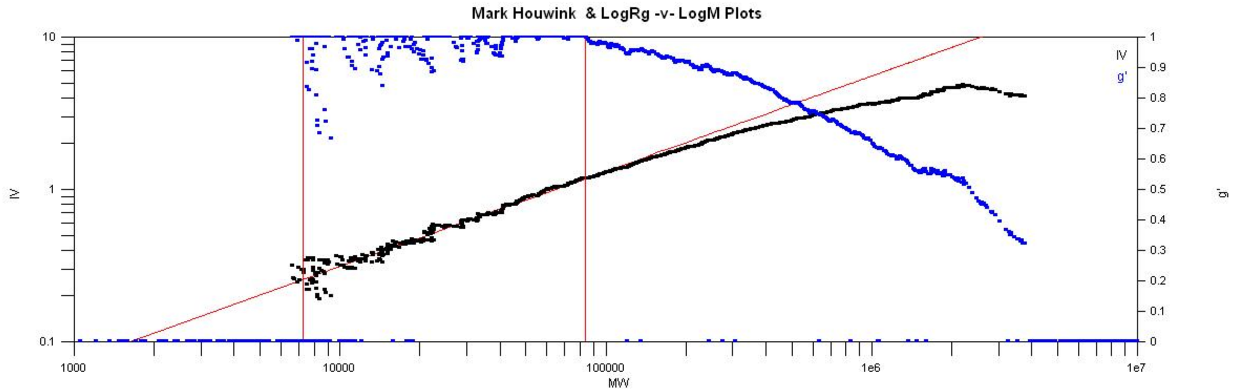

Figure S22: Diagram of IV and g' vs log Mw for LDPE

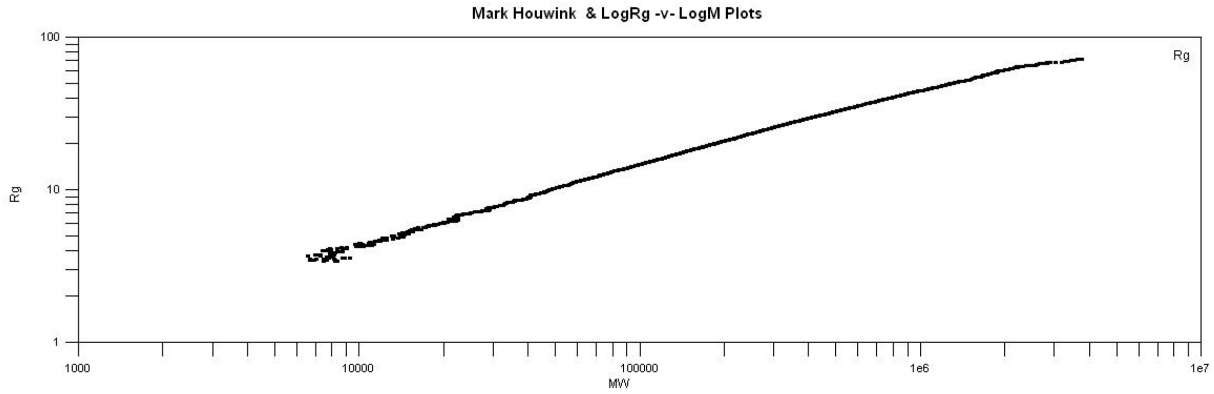

Figure S23: Diagram of Rg vs log Mw for LDPE

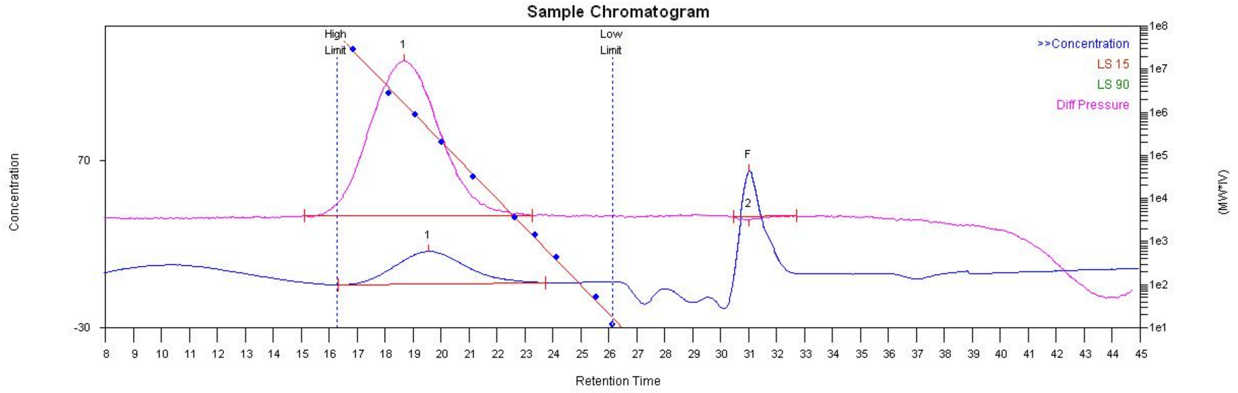

Figure S24: GPC chromatogram of PP

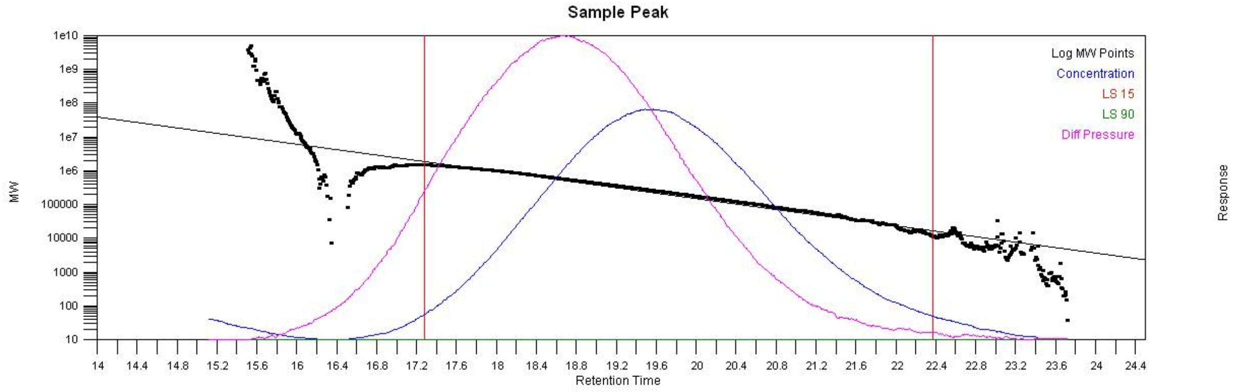

Figure S25: Diagram of log Mw vs Retention Time for PP

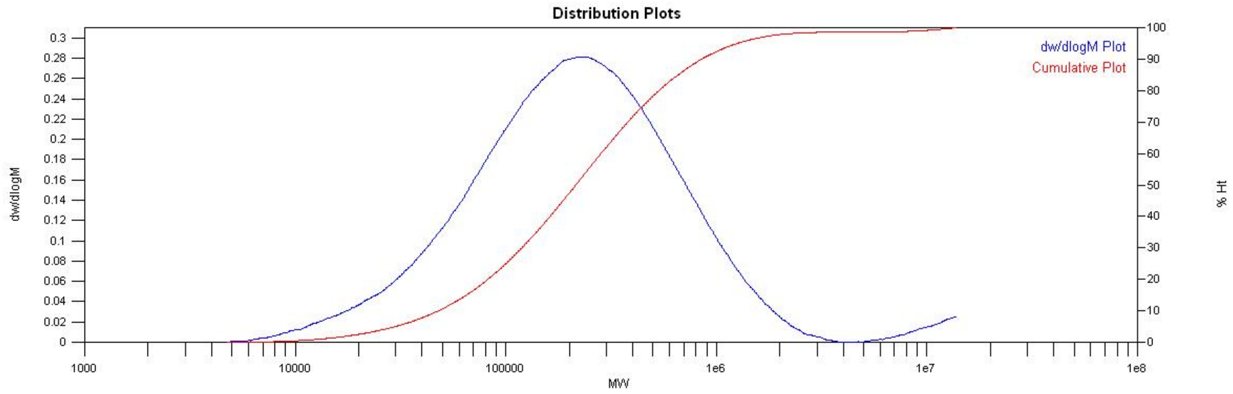

Figure S26: Diagram of dw/dlogM vs log Mw for PP

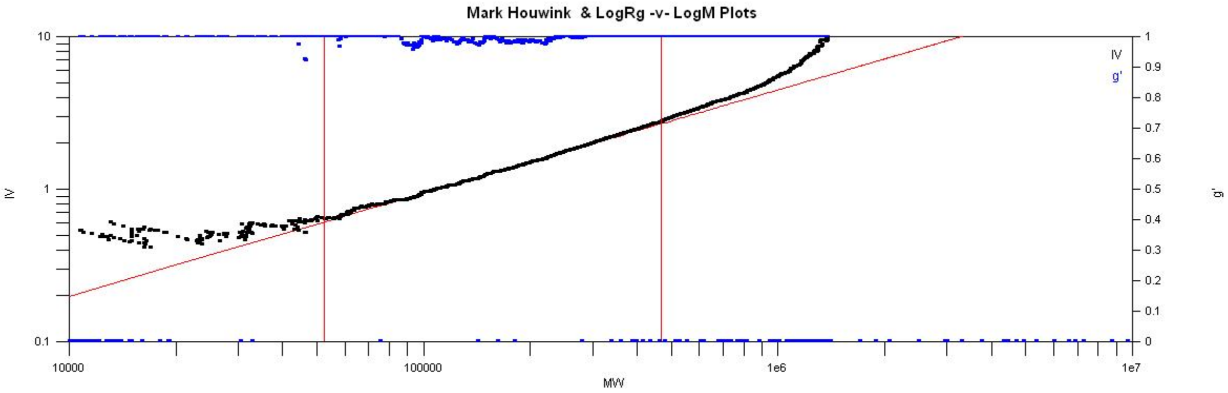

Figure S27: Diagram of IV and g' vs log Mw for PP

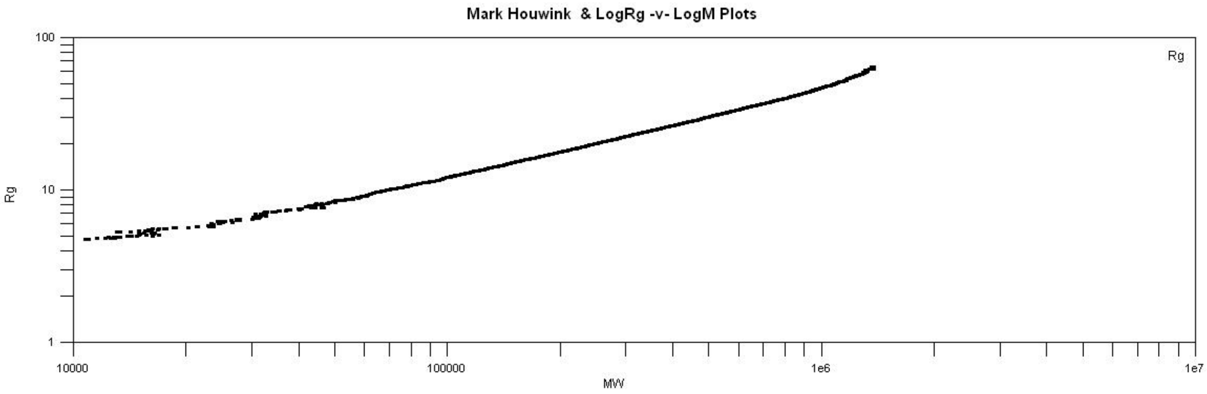

Figure S28: Diagram of Rg vs log Mw for PP

Table S1: Average molecular weight, PDI, weight fraction, and bulk intrinsic viscosity of samples obtained by GPC analysis#

	**HDPE**	**LLDPE**	**LDPE**	**PP**
M_p	340620	96195	177110	152954
M_n	17667	44710	38573	72129
M_w	497526	190152	254206	289255
M_z	2340348	610487	827577	2047375
M_{z+1}	4419086	1204601	1782909	5379474
Dispersity	28.161	4.253	6.59026	4.01022
1000000-7000000 %	14.67	2.61679	4.29641	2.6503
100000-1000000 %	37.67	45.7568	52.135	58.7505
1000-100000 %	47.22	51.6265	43.5627	38.3098
Bulk Intrinsic Viscosity (dL/g)	4.6645	3.0178	1.5927	2.224
